# Emerging evidence from a systematic review of safety of pre‐exposure prophylaxis for pregnant and postpartum women: where are we now and where are we heading?

**DOI:** 10.1002/jia2.25426

**Published:** 2020-01-08

**Authors:** Dvora L Joseph Davey, Jillian Pintye, Jared M Baeten, Grace Aldrovandi, Rachel Baggaley, Linda‐Gail Bekker, Connie Celum, Benjamin H Chi, Thomas J Coates, Jessica E Haberer, Renee Heffron, John Kinuthia, Lynn T Matthews, James McIntyre, Dhayendre Moodley, Lynne M Mofenson, Nelly Mugo, Landon Myer, Andrew Mujugira, Steven Shoptaw, Lynda Stranix‐Chibanda, Grace John‐Stewart, for the PrEP in Pregnancy Working Group

**Affiliations:** ^1^ Department of Epidemiology Fielding School of Public Health University of California Los Angeles CA USA; ^2^ Division of Epidemiology and Biostatistics School of Public Health and Family Medicine University of Cape Town Cape Town South Africa; ^3^ Department of Global Health University of Washington Seattle WA USA; ^4^ Department of Epidemiology University of Washington Seattle WA USA; ^5^ Department of Medicine University of Washington Seattle WA USA; ^6^ Geffen School of Medicine University of California Los Angeles CA USA; ^7^ World Health Organization Geneva Switzerland; ^8^ Faculty of Health Sciences Desmond Tutu HIV Centre Institute of Infectious Disease and Molecular Medicine University of Cape Town Cape Town NC South Africa; ^9^ Department of Obstetrics and Gynecology University of North Carolina at Chapel Hill Chapel Hill NC USA; ^10^ Center for Global Health Massachusetts General Hospital Boston MA USA; ^11^ Department of Research and Programs Kenyatta National Hospital Nairobi Kenya; ^12^ Department of Medicine University of Alabama Birmingham AL USA; ^13^ ANOVA Johannesburg South Africa; ^14^ Department of Obstetrics and Gynaecology University of KwaZulu‐Natal Durban South Africa; ^15^ Centre for AIDS Research in South Africa Durban South Africa; ^16^ Elizabeth Glaser Pediatric AIDS Foundation Washington DC USA; ^17^ Center for Clinical Research Kenya Medical Research Institute (KEMRI) Nairobi Kenya; ^18^ Infectious Diseases Institute Makerere University Kampala Uganda; ^19^ Department of Family Medicine University of California Los Angeles CA USA; ^20^ University of Zimbabwe College of Health Sciences Harare Zimbabwe

**Keywords:** preexposure prophylaxis, PrEP, pregnancy, breastfeeding, PMTCT, prevention of mother to child transmission, HIV

## Abstract

**Introduction:**

HIV incidence is high during pregnancy and breastfeeding with HIV acquisition risk more than doubling during pregnancy and the postpartum period compared to when women are not pregnant. The World Health Organization recommends offering pre‐exposure prophylaxis (PrEP) to pregnant and postpartum women at substantial risk of HIV infection. However, maternal PrEP national guidelines differ and most countries with high maternal HIV incidence are not offering PrEP. We conducted a systematic review of recent research on PrEP safety in pregnancy to inform national policy and rollout.

**Methods:**

We used a standard Preferred Reporting Items for Systematic Reviews and Meta‐Analysis (PRISMA) approach to conduct a systematic review by searching for completed, ongoing, or planned PrEP in pregnancy projects or studies from http://www.clinicaltrials.gov, PubMed and NIH RePORTER from 2014 to March 2019. We performed a systematic review of studies that assess tenofovir disoproxil fumarate (TDF)‐based oral PrEP safety in pregnant and breastfeeding HIV‐uninfected women.

**Results and discussion:**

We identified 14 completed (n = 5) and ongoing/planned (n = 9) studies that evaluate maternal and/or infant outcomes following PrEP exposure during pregnancy or breastfeeding. None of the completed studies found differences in pregnancy or perinatal outcomes associated with PrEP exposure. Nine ongoing studies, to be completed by 2022, will provide data on >6200 additional PrEP‐exposed pregnancies and assess perinatal, infant growth and bone health outcomes, expanding by sixfold the data on PrEP safety in pregnancy. Research gaps include limited data on (1) accurately measured PrEP exposure within maternal and infant populations including drug levels needed for maternal protection; (2) uncommon perinatal outcomes (e.g. congenital anomalies); (3) infant outcomes such as bone growth beyond one year following PrEP exposure; (4) outcomes in HIV‐uninfected women who use PrEP during pregnancy and/or lactation.

**Conclusions:**

Expanding delivery of PrEP is an essential strategy to reduce HIV incidence in pregnancy and breastfeeding women. Early safety studies of PrEP among pregnant women without HIV infection are reassuring and ongoing/planned studies will contribute extensive new data to bolster the safety profile of PrEP use in pregnancy. However, addressing research gaps is essential to expanding PrEP delivery for women in the context of pregnancy.

## 1 Introduction

HIV incidence is high during pregnancy and breastfeeding 1 with HIV acquisition risk more than doubling during pregnancy and the postpartum period compared to when women are not pregnant 2. Risk of HIV acquisition during pregnancy and postpartum translates to a substantial cumulative period of risk over the course of women's lives in sub‐Saharan African regions where both fertility and HIV prevalence are high 1. Additionally, acute maternal HIV infection during pregnancy and breastfeeding threatens maternal health and increases the risk of vertical transmission accounting for an estimated 30% of new HIV infections in some settings 3, 4, 5. Globally 160,000 infants and children were newly infected with HIV in 2018, mostly in low‐ and middle‐income countries (LMICs) 6. The World Health Organization (WHO) recommends offering tenofovir (TFV) disoproxil fumarate (TDF)‐based oral pre‐exposure prophylaxis (PrEP) to pregnant and postpartum women at substantial risk of HIV acquisition 7. Although TDF is available as part of antiretroviral treatment (ART) regimens for pregnant women living with HIV, the availability of TDF‐based PrEP for HIV‐uninfected mothers remains limited in many of these settings 8.

Expanding delivery of PrEP to pregnant women at high risk of HIV is an important prevention strategy, yet national guidelines differ substantially in recommending PrEP use during pregnancy and lactation 8. Programmatic PrEP delivery to pregnant women is ongoing in Kenya 9. Guidelines in Zimbabwe 10, Swaziland, Australia, New Zealand, Canada and US 11 are permissive of PrEP use during pregnancy 8. Uganda and Botswana have policies that recommend or allow PrEP for discordant couples trying to conceive 8. In contrast, the desire for additional safety data on maternal PrEP use has hindered PrEP implementation among pregnant women in South Africa, Malawi, Zambia and other HIV high‐burden settings 8. Until recently the South African Department of Health did not recommended PrEP for pregnant women 12, despite modelling studies demonstrating the important potential influence on eliminating maternal‐to‐child transmission of HIV (eMTCT) 13, stating that it was medically “contraindicated” in programmatic delivery. However, PrEP use among pregnant and breastfeeding women has been permitted in the context of clinical trials 14, 15, 16, 17.

Studies of infants exposed to antiretroviral therapy (ART) for treatment of maternal HIV infection, some ongoing for decades, provide critical information about TDF‐containing ART exposure and birth, bone and growth outcomes 18. A systematic review commissioned by WHO in 2016 included 33 studies and evaluated the safety of TDF use in pregnancy and breastfeeding, relying primarily on studies among women living with HIV on TDF‐containing combination antiretroviral therapy (ART) or hepatitis B virus (HBV)‐infected women on TDF 18. The review concluded that there was no safety‐related rationale for prohibiting oral TDF‐based PrEP use during pregnancy and lactation. However, it is difficult to disentangle the impact of concomitant ART drugs and HIV disease on TDF safety data in women living with HIV 19. The results of studies from women living with HIV may not reflect safety of PrEP use among HIV‐uninfected mothers. Additionally, the acceptable risk‐benefit ratio may differ for HIV treatment versus prevention in pregnant women. As a preventative agent for women without HIV infection, safety of PrEP must necessarily be high, and the WHO, the US National Institutes of Health and the Ministry of Health in Kenya have called for more detailed and longer‐term safety evaluations of PrEP‐exposed children and women to inform clinical guidelines and overcome regulatory barriers to scale‐up 7, 15, 16, 20.

We performed a systematic review of completed and ongoing studies that assess PrEP safety among pregnant and breastfeeding HIV‐uninfected women and their infants to synthesize available PrEP‐specific data and complement previous TDF‐focused reviews 18, identify gaps for further safety research, and inform the development of clinical guidelines for PrEP use in pregnancy.

## 2 Methods

This review focuses on research among HIV‐uninfected women exposed to PrEP during pregnancy and/or the postpartum period, and complements previous reviews on TDF safety that predominantly included women living with HIV who were exposed to TDF within ART regimens 18, 21. We focused on studies that included maternal and infant outcomes following PrEP exposure during pregnancy and breastfeeding. We included studies that had been published, or were in progress, between January 2014 and March 2019. Maternal and infant outcomes included gestational age at birth, birth weight/length, pregnancy outcomes and complications, congenital anomalies, infant growth and bone health indicators, maternal adverse events and metabolic changes.

### 2.1 Search strategy and selection criteria

We used a standard Preferred Reporting Items for Systematic Reviews and Meta‐Analysis (PRISMA) approach 22 to conduct a systematic review by searching for completed, ongoing, or planned PrEP in pregnancy projects or studies on http://www.clinicaltrials.gov, PubMed and NIH RePORTER using the following search terms: HIV pre‐exposure prophylaxis; preexposure prophylaxis; PrEP; tenofovir disoproxil fumarate; pregnancy; postpartum; and breastfeeding. We limited the search terms to PrEP key words to only include studies among HIV‐uninfected women, and not HIV‐infected pregnant women on TFV, to intentionally capture the most up‐to‐date PrEP in pregnancy projects and complement prior reviews that sought to assess TDF safety more broadly in HIV‐infected and HBV‐infected women 18, 21. We included all studies that reported providing oral PrEP to pregnant and/or breastfeeding mothers in any year and in any geographic location. We excluded studies that: (1) did not evaluate maternal or infant outcomes (e.g. gestational age at birth, birth weight/length, pregnancy outcomes and complications, congenital anomalies, infant growth and bone health indicators, maternal adverse events and metabolic changes) following oral PrEP use (e.g. commentary/review papers, formative or qualitative studies, evaluations of provider knowledge, etc.); (2) provided non‐oral PrEP agents (e.g. vaginal microbicides, rings, etc.); and (3) excluded pregnant or breastfeeding women. Two reviewers (DJD and JP) independently assessed the papers, study descriptions and protocols to determine whether they should be included. The same two reviewers analysed the studies to determine if the study was ongoing or completed and what the study exposures and outcomes were. If there were any disagreements, they were resolved by a third party (GJS). DJD sent out the data to study investigators to ensure that the information was correct and to collect any additional information.

## 3 Results and discussion

**Figure 1:**
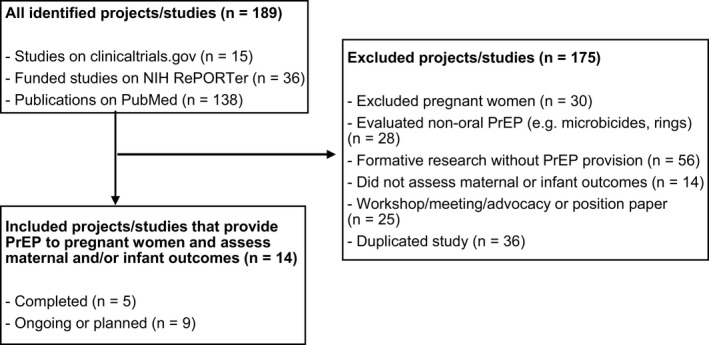
PRISMA flow chart of identified studies and projects on maternal and infant outcomes following oral PrEP exposure during pregnancy and breastfeeding (10 to 30 March 2019).

We identified 189 studies or publications that included HIV pre‐exposure prophylaxis; preexposure prophylaxis; PrEP; pregnancy; postpartum; and breastfeeding in their search terms. After removal of duplicated studies (n = 36); or those that did not meet eligibility criteria (n = 139), we included a total of 14 studies (Figure 1). Of those studies, five were completed, while nine studies were ongoing or planned.

### 3.1 Completed PrEP in pregnancy studies

The five completed studies included women in Kenya, Uganda, Zimbabwe and South Africa between 2014 and 2018 23, 24, 25, 26, 27, 28. These studies included a total of 1042 PrEP‐exposed pregnancies. Four of the five completed studies found no differences in pregnancy or infant outcomes in the PrEP‐exposed compared to unexposed arms. One study found that PrEP exposed infants had a lower z‐score for length at 1‐month; however, there was no difference at 1‐year 25. Most of the studies were sub‐studies of PrEP randomized control trials among HIV‐serodiscordant couples or young women in African settings, including the Partners PrEP Study 23, FEM‐PrEP 24 and VOICE.

**Figure 2:**
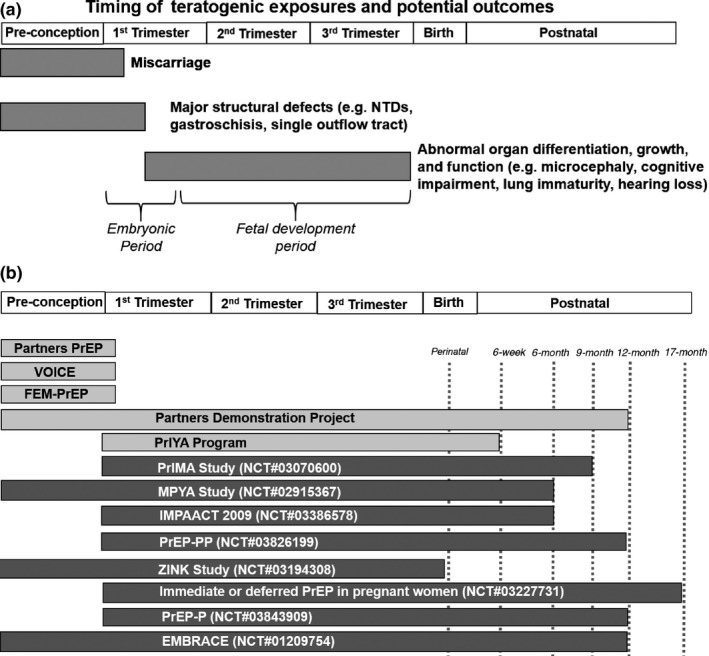
(a) Timing of teratogenic exposures during pregnancy and adverse potential outcomes 64. (b) Time and duration of pre/postnatal PrEP exposure in completed (light grey) and ongoing/planned studies (dark grey).

The original PrEP efficacy trials excluded pregnant women from enrolment and conducted monthly pregnancy testing, with discontinuation of study drug as soon as pregnancy was detected. Thus, they provide data based on early first trimester exposure, when teratogenic exposures can cause pregnancy loss and structural abnormalities such as neural tube defects (Figure 2a,b); no differences in perinatal outcomes were detected between exposure groups 24, 25, 29.

Maternal and/or infant exposure to TDF/FTC during pregnancy was not quantified using drug level testing in any PrEP in pregnancy safety study to date and TDF/FTC drug levels may vary by when PrEP use started in pregnancy, duration of PrEP use in pregnancy and breastfeeding and maternal adherence (Table 1). A recently completed evaluation of programmatic PrEP delivery via routine ante‐/post‐natal clinics in Kenya (the PrEP Implementation for Young Women and Adolescents [PrIYA] Programme) found no association between PrEP use during pregnancy and early infant outcomes including gestational age at birth, birth length/weight and growth indicators at six weeks 30. Infants were evaluated at six weeks in n = 246 pregnant women. The Partners Demonstration Project enrolled 30 women in who elected to continue PrEP after becoming pregnant found that PrEP‐exposed infants had a lower z‐score for length at 1‐month, but there was no difference at 1‐year follow‐up 25.

**Table 1:** Completed projects and studies evaluating maternal and infant outcomes following PrEP exposure during pregnancy and breastfeeding (n = 5)

Study; Study Location; Lead author; Year	Design & Population	PrEP‐exposed pregnancies; time exposed	Pregnancy outcomes	Infant outcomes
Partners PrEP Study; Kenya and Uganda; Mugo; 2014 23	Randomized trial in serodiscordant couples; 431 pregnancies	n = 335; Median duration of gestation at time of pregnancy detection = 37 days (IQR 29–46) for tenofovir disoproxil fumarate and 35 days (IQR 29–42) for emtricitabine/tenofovir disoproxil fumarate	No difference in pregnancy loss (31% in PrEP exposed vs. 32% in placebo; *p* = 0.46) or preterm birth (3.4% in PrEP exposed vs. 7.7% in placebo; *p* = 0.16)	No difference in congenital anomalies, growth at 1‐year (5% in TDF using women vs. 7.6% in placebo; *p* = 0.51)
FEM‐PrEP; Kenya, South Africa and Kenya; Callahan; 2015 24	Randomized, placebo‐controlled trial; 115 pregnancies	n = 69; Pregnancy tests were performed monthly and, if positive, study product was withheld	Of 115 women who became pregnant during the study, 30 (26%) reported outcome data with no difference by study arm (data by arm not reported)	None reported
VOICE; Uganda, South Africa and Zimbabwe; Bunge; 2015 29	Randomized trial; 452 pregnancies	n = 263; Pregnancy tests were performed monthly and, if positive, study product was withheld	Early pregnancy loss was not higher among women exposed to TDF‐containing PrEP compared to placebo	None reported
Partners Demonstration Project; Kenya and Uganda; Heffron; 2018 25	Demonstration project in serodiscordant couples; 126 pregnancies	n = 30; Women were dispensed PrEP a median of six months (IQR 4–8) during pregnancy; 52% of women took at least 80% of expected doses.	No difference in pregnancy loss (17% in PrEP‐exposed vs. 24% in control; *p* = 0.7) or preterm birth (0% vs. 8%; *p* = 0.4) by PrEP use in pregnancy	PrEP exposed infants lower z‐score for length at 1‐month; no difference at 1‐year
PrIYA Programme; Kenya; Dettinger; 2018 26, 62	Implementation programme; 4680 pregnancies included in safety evaluation	n = 246; 47% initiated PrEP in the second trimester, and 41% reported using PrEP for 1‐three months during pregnancy	Rates of reported preterm birth were similar between the two groups (3.1% PrEP exposed, 4.2% non‐PrEP, *p* = 0.50), and birthweight (median 3.3 kg in both groups, *p* = 0.14).	No differences in small for gestational age (1.9% vs 6.7%, *p* = 0.18); or congenital malformations (n = 0 in PrEP exposed vs.13 reported in PrEP unexposed). No differences by groun in weight‐for‐age, length‐for‐age, or weight‐for‐length Z‐scores (*p* = 0.60, 0.13, 0.76 respectively).

### 3.2 Ongoing or planned PrEP in pregnancy studies

We identified nine ongoing or planned studies that evaluate pregnancy and infant outcomes following PrEP exposure during pregnancy and/or breastfeeding. These studies are being conducted at single‐ or multi‐country sites across Kenya (n = 2), Malawi (n = 1), South Africa (n = 5), Uganda (n = 2), United States (n = 1), Zimbabwe (n = 1) and one International Registry. The cumulative number of anticipated PrEP‐exposed pregnancies is approximately 6194 women, with planned enrolment in individual studies ranging from 40 to 4500 pregnant women. In South Africa, an ongoing open‐label randomized controlled trial of pregnant women is comparing pregnancy and infant outcomes, maternal and infant bone health and infant growth over an 18‐month period between women assigned to initiate PrEP in the second trimester of pregnancy versus delayed PrEP initiation at cessation of breastfeeding (CAP016; NCT#03227731). One ongoing cluster‐randomized trial in Kenya, the PrEP Implementation for Mothers in Antenatal Care (PrIMA) Study (NCT#03070600), compares two approaches for programmatically delivering PrEP within routine antenatal care clinics (universal PrEP‐offer versus targeted‐offer based on an empiric HIV risk score). Another planned randomized trial (PrEP‐P) is a US‐based pharmacologic trial of TDF/FTC dosing which will compare 1 tablet of 300 mg TDF/200 mg FTC per day versus a pregnancy‐adjusted 2 tablets of 300 mg TDF/200 mg FTC per day that will assess birth outcomes between arms (NCT#03834909).

The remaining six studies are non‐randomized observational studies (Table 2). IMPAACT 2009 (NCT#03386578) is a parallel observational cohort study that is enrolling 350 women in South Africa, Malawi, Zimbabwe and Uganda to evaluate enhanced adherence support messaging and feedback from counsellors on PrEP adherence. The PrEP in pregnancy and postpartum (PrEP‐PP) study (NCT#03902418) in Cape Town is enrolling 1200 pregnant women at first antenatal care visit in an open cohort and follow them up over time to evaluate PrEP uptake, adherence (via dried blood spot [DBS] analysis of TFV) and contributing factors to PrEP uptake and adherence. The Monitoring PrEP in Young Adult women (MPYA) study is evaluating patterns of PrEP use and adherence among 350 women in Kenya using a real‐time electronic monitor, as well as DBS in a subset of participants. The ZINK study in Durban, South Africa is assessing PrEP use as part of a safer conception package in 350 HIV‐uninfected women who are planning to become pregnant with an HIV‐seropositive or unknown‐serostatus partner 31. These studies are evaluating adverse events in women who take PrEP versus those who do not take PrEP during pregnancy and the lactation period and will compare adverse pregnancy and infant health outcomes (e.g. spontaneous abortion, stillbirth, preterm delivery or small for gestational age and frequency of infant death). Finally, the Evaluation of Maternal and Baby Outcome Registry After Chemoprophylactic Exposure study (EMBRACE) is evaluating maternal and infant outcomes in 550 women who participated in a PrEP or microbicide trial and became pregnant (NCT#01209754). The international Antiretroviral Pregnancy Registry (APR) is an international monitoring registry that prospectively monitors antiretroviral‐exposed pregnancies that are reported to the APR for birth outcomes, including infants born to women receiving TFV‐containing ART regimens and those receiving TDF/FTC for PrEP (NCT#01865786) 32.

**Table 2:** Ongoing or planned projects and studies evaluating maternal and infant outcomes following PrEP exposure during pregnancy and breastfeeding (n = 9)

Study; Country; Lead researcher(s)	Design & Population	Exposure (n = planned exposed pregnancies); intervention	Planned measurement of prenatal or perinatal outcomes	Planned measurement of postnatal outcomes
PrIMA Study; Kenya; John‐Stewart, Baeten 63	Cluster randomized trial; (anticipated) 4000 pregnant women attending ANC in Kenya	Anticipated 20% PrEP uptake; n = 3749 women enrolled in pregnancy (as of January 2019)	Pregnancy loss, gestational age at birth, birth length, birth weight, neonatal death and congenital abnormalities	Growth indicators at six weeks, fouteen weeks, six months and nine months to be assessed
Monitoring PrEP in Young Adult women (MPYA): Kenya; Haberer, Baeten, Bukusi, Mugo	Observational cohort with nested randomized controlled trial of SMS reminders in 350 women (92 pregnancies as of June 2019)	Determine patterns of PrEP use, the impact of SMS reminders on adherence, and technical function, acceptability, cost, and validity of Wisepill device in n = 350 women	Gestational age at birth, birth weight, pregnancy outcomes (ectopic, pregnancy loss, live birth), pregnancy complications (pre‐eclampsia, gestational diabetes, febrile illness, rash)	Congenital abnormalities, acute illness, infant death
IMPAACT 2009 (PrEP comparison); Zimbabwe, Uganda, South Africa and Malawi; Chi & Stranix‐Chibanda (NCT#03386578)	Parallel observational cohort study; 350 women to achieve 300 women	Enhanced adherence support, including SMS messaging and feedback of drug levels with tailored counselling.	Chemistry abnormalities or higher AEs, composite outcome of adverse pregnancy outcomes (spontaneous abortion, stillbirth (≥20 weeks gestation), preterm delivery (<37 weeks), or small for gestational age and infant death	Grade 3 or higher adverse events (through week 26), maternal BMD (DXA of lumbar spine and hip); infant bone mineral content (DXA scans and whole body and lumbar spines), infant creatinine levels, creatinine clearance, length for age z‐score
Evaluating PrEP cascade in pregnant and postpartum women (PrEP‐PP); South Africa; Coates, Myer & Joseph Davey (NCT#03902481)	Observational single arm; 1200 pregnant women in ANC	Estimated PrEP uptake = 60% (n = 720 exposed pregnancies)	Pregnancy loss, gestational age and size, and adverse events	Growth indicators at birth, three, six, nine, twelve months, congenital abnormalities, acute illness, infant death
Safer Conception for Women: PrEP Uptake/Adherence to Reduce Peri‐conception HIV Risk for South African Women (ZINK study); South Africa; Matthews & Smit 31 (NCT#03194308)	Observational single arm of 350 women expecting to get pregnant	200 women enrolled (March 2019); PrEP uptake approximately 60%, pregnancy prevalence at 6‐months = 31%	Gestational age at birth, birth length, birth weight, pregnancy loss and congenital abnormalities	None reported
Safety of PrEP in pregnant and breastfeeding women: a RCT of immediate vs deferred PrEP in pregnant and breastfeeding women; South Africa; Moodley (NCT#03227732)	An Open label Randomized controlled trial of 842 pregnant women	Arm A = exposure to TDF/FTC vs Arm B = no exposure to TDF/FTC during pregnancy and breastfeeding (delayed PrEP initiation)	Preterm birth, low birth weight (<2500g), pregnancy loss, small for gestational age, neonatal death, and maternal kidney function in relation to actual exposure to TFV (DBS)	Bone mineral density in mothers and bone mineral content in infants at six weeks post‐delivery and 6‐monthly until week 74 in relation to duration of breastfeeding and actual exposure to TFV (in utero and breastfeeding).. Infant growth parameters until 74 weeks in relation TFV exposure during breastfeeding. Maternal and infant safety assessments at 6 monthly intervals until 74 weeks in relation to actual exposure to TDF/FTC (TFV levels in DBS).
Pre‐Exposure Prophylaxis Dosing in Pregnancy to Optimize HIV Prevention (PREP‐P); USA (DC, Baltimore); Hendrix (NCT#03843909)	Randomized trial (n = 40 anticipated) to one of two parallel study arms, involving dosing of TDF/FTC (1 tablet/day vs. 2 tablets/day)	Randomized trial of 2 PrEP pharmacokinetic (PK) dosing regimens from 1st trimester to 12 weeks following delivery (postpartum) to evaluate PK, safety for maternal and fetal/infant safety signals, and adherence.	Maternal safety assessments (changes in kidney function and blood flow) Ultrasound and biophysical profiles at 2nd and 3rd trimester interval growth ultrasound	Maternal safety assessments (changes in kidney function and blood flow) until six months postpartum. Mitochondrial function at birth; DXA scans at three to six, twenty‐four to twenty‐eight and fifty to fifty‐four weeks of age; kidney function by blood sample at three to six weeks and 24 to 28 weeks.
EMBRACE (Evaluation of Maternal and Baby Outcome Registry After Chemoprophylactic Exposure) (EMBRACE); South Africa, Uganda and Zimbabwe; Beigi (Study Chair), MTN (NCT#01209754)	Prospective observational cohort investigation of exposures to study agents under investigation for HIV prevention.	N = 550 pregnant participants (and 400 live infants) who became pregnant during their participation in a microbicide or PrEP trial, or who have had planned exposures in pregnancy safety studies & babies resulting from these pregnancies.	Preterm birth, stillbirth or intrauterine fetal demise, ectopic pregnancy, spontaneous abortion, intrapartum & postpartum hemorrhage, non‐ reassuring fetal status, chorioamnionitis, hypertensive disorders, gestational diabetes and intrauterine growth restriction	Malformations identified in the first year of life; growth parameters (weight, length, and head circumference at birth, one month, six months and 12 months) in the first year of life
A Prospective, Observational Study of Pregnancy Outcomes Among Women Exposed to Truvada for PrEP Indication; International; 2018 (NCT#01865786) 32	Prospective observational cohort to describe pregnancy outcomes nested in the Antiretroviral Pregnancy Registry	n = 99 participants; time exposed unknown *(results not yet published)*	Pregnancy outcomes (not specified)	Presence of congenital malformations

### 3.3 Research gaps and opportunities

Despite the number of ongoing and planned studies, some gaps remain, including limited data on (1) accurately measured PrEP exposure within maternal and infant populations including drug levels needed for optimal protection; (2) uncommon perinatal outcomes such as congenital anomalies; (3) outcomes such as bone growth beyond one year in infants exposed to PrEP during pregnancy and/or lactation; (4) outcomes in HIV‐uninfected women who use PrEP during pregnancy and/or lactation. Below we describe these identified gaps and opportunities for potential research (Table 3).

**Table 3:** Gaps and opportunities in research on safety of PrEP during pregnancy and breastfeeding

	Gaps	Opportunities
PrEP exposure within maternal and infant populations	Evaluation of prevention‐effective thresholds of PrEP exposure in pregnancy	Quantify direct infant exposure using infant biomarkers To understand PrEP effectiveness thresholds, would require a study of HIV incidence at different thresholds of PrEP (which may not be possible)
Perinatal outcomes that occur uncommonly	Studies under powered to evaluate uncommon perinatal outcomes such as congenital anomalies Most studies include women who initiate PrEP while pregnant and do not evaluate PrEP exposure during the peri‐conception period when major organ development occurs Only one study used ultrasound to assess gestational age	Combine data across studies to improve statistical power to evaluate uncommon pregnancy and infant outcomes Evaluate PrEP use during peri‐conception and very early pregnancy Gold standard measurement (e.g. antenatal ultrasound for gestational age) to provide high quality safety data
Outcomes beyond infancy among PrEP‐exposed children	Few studies evaluate longer‐term infant outcomes following prenatal PrEP exposure Limited studies on outcomes such as bone growth beyond one year	Evaluate longer‐term outcomes beyond infancy and into early childhood following prenatal PrEP exposure Incorporate longer term measures of infant bone growth
Outcomes among HIV‐uninfected women who use PrEP during pregnancy and/or lactation	Limited maternal‐specific outcomes are included in current studies except for monitoring renal and liver function post‐PrEP initiation Analysis of STIs in pregnant women on PrEP	Studies that include robust evaluation of mother‐focused safety outcomes beyond pregnancy/infant outcomes Studies that evaluate STIs among maternal PrEP users

#### 3.3.1 PrEP exposure within maternal and infant populations

Pregnancy can affect drug pharmacokinetics. Only one small study previously examined PrEP pharmacokinetics during pregnancy and found that plasma TFV and intracellular TFV‐DP in DBS were 45–58% lower during pregnancy compared with non‐pregnant periods after adjusting for adherence as measured by Medication Event Monitoring System (MEMS) openings 33. Differences in drug concentrations were generally greater in the second and third trimester than in early pregnancy, consistent with known physiologic changes that occur during pregnancy that have the potential to affect drug pharmacokinetics, including changes in gastrointestinal absorption, renal excretion, hepatic metabolic enzymes and overall body water distribution 34, 35. Prevention‐effective thresholds of PrEP exposure among African women have not been determined to date, and physiological changes during pregnancy may further obfuscate protective thresholds for pregnant women. It is uncertain whether lower drug levels during pregnancy have implications for protection against HIV among pregnant women receiving PrEP. All ongoing/planned studies we identified will measure HIV incidence among PrEP‐using pregnant and postpartum women, though none are powered to detect significant differences in this important outcome. Two studies identified in our review will investigate pharmacokinetics of PrEP in pregnancy to establish reference thresholds for prevention‐effective adherence for pregnant women using DBS, plasma and peripheral blood mononuclear cells (PBMCs) (IMPAACT 2009 and PrEP Dosing in Pregnancy to Optimize HIV Prevention studies). Additional studies among larger populations are needed to understand how pregnancy may affect prevention‐effective PrEP exposure in this population. As more options beyond oral TDF‐based PrEP become available, future studies are critical to examine bioavailability and metabolism of new PrEP agents among pregnant women to fully understand implications of pregnancy on prevention‐effective PrEP exposure.

Currently oral TDF/FTC is the only medication with a label indication as PrEP. Tenofovir alafenamide (TAF), a novel lower dose prodrug for TFV, delivers 90% lower plasma TFV concentrations with higher intracellular TFV‐DP levels; the lower plasma levels are expected to result in less bone demineralization and renal toxicity. TAF recently received US FDA approval for HIV treatment and results of the DISCOVER trials which demonstrated non‐inferiority of F‐TAF and FTC‐TDF TAF for PrEP among men who have sex with men has been submitted to the FDA (http://www.clinicaltrials.gov #NCT02842086) 36. TAF has not been studied for PrEP in young women although PK and PD studies are underway. Therefore, safety data on prenatal TAF use will likely accumulate over time as TAF use as part of HIV treatment regimens expands among Women living with HIV of reproductive age. Unlike TDF, which had a long history of being used for HIV treatment among pregnant women prior to PrEP. TAF as PrEP and newer agents will take considerable time to accrue meaningful data on prenatal use. Given the trajectory to establish efficacy and obtain regulatory approvals, TAF as PrEP may take years to reach African populations and pregnant women. Thus, advancing safety evidence for TDF‐based PrEP use in pregnancy will be immediately informative as TDF is available now and data on TAF as PrEP are unavailable in women, including pregnant women.

Although the clinical significance of the observed differences in DBS TFV‐DP levels during pregnancy is unclear, this needs to be considered in interpretation of results from PrEP safety studies which will quantify maternal PrEP adherence using DBS TFV‐DP levels and/or plasma TFV levels. Of the ongoing or planned PrEP in pregnancy studies, four studies include an objective measurement of maternal PrEP exposure during pregnancy and/or breastfeeding either in the entire cohort or among a subset. TFV‐DP levels in DBS will be evaluated to determine maternal PrEP adherence and quantify TFV‐DP concentrations in the pharmacokinetic phase of IMPAACT 2009 (under directly observed treatment conditions), PrIMA, the HPP study, PrEP‐PP and ZINK; MPYA will report electronic adherence data, as well as DBS in a subset of participants. IMPAACT 2009 will also evaluate cord blood plasma TFV‐DP concentrations (up to 14 days post‐delivery). Ongoing and future studies need to account for lower DBS TFV‐DP levels during pregnancy when quantifying and defining PrEP exposure and adherence in maternal populations. When IMPAACT 2009 results are available, they could potentially serve as standardized DBS TFV‐DP thresholds for PrEP exposure in pregnancy to understand optimal adherence levels, but will be underpowered to detect protection thresholds. Future studies could incorporate evaluation of PrEP exposure using other non‐invasive biomarkers that may be less affected by pregnancy, such as TFV hair or urine levels, in parallel with other matrices. Hair ARV levels in women living with HIV strongly predict virologic outcomes during pregnancy, suggesting hair is an accurate biomarker for ARV exposure in pregnancy 37.

None of the completed, ongoing or planned studies identified to date will quantify direct infant exposure using infant biomarkers. Infant biomarkers provide precise measurement of infant exposure and are useful for interpreting safety data. Infant exposure to maternal drugs is commonly estimated as a percentage of the maternal dose. However, it is difficult to accurately determine foetal and infant ARV drug exposure based on maternal drug dosage due to variations in maternal adherence, pharmacokinetics, placental transfer, and foetal/infant metabolism. ARV concentrations in cord blood reflect maternal exposure over a short time period just prior to delivery and do not accurately represent exposure in a newborn already capable of drug metabolism 38, 39, 40, 41. Similarly, ARV levels in meconium measure short‐term exposure and collection can be logistically challenging 42, 43 While there is minimal penetration of TFV (the metabolite of TDF) into breastmilk, there is significant penetration of FTC, though breastmilk concentrations do not incorporate the effects of infant absorption and maturing pathways of infant metabolism in terms of drug exposure to the infant 44, 45, 46, 47. Given these limitations, few studies to date have been able to accurately quantify cumulative exposure to ARVs in the infant during pregnancy and breastfeeding, and only one has evaluated infant exposure to TDF/FTC used for PrEP in breastfeeding mothers 47. One prior study evaluated hair levels of mother and infant ARVs as a metric of drug transfer in the perinatal and breastfeeding periods, though TFV was not assessed 48. Future studies may consider similar approaches for quantifying PrEP exposure *in utero* through breastfeeding cessation to gain a more accurate measurement of infant PrEP exposure following maternal PrEP use in pregnancy and breastfeeding.

#### 3.3.2 Perinatal outcomes that occur uncommonly

Most of the studies we identified are insufficiently powered to detect differences in uncommon maternal and infant outcomes such as congenital anomalies. Combining data across studies may provide more statistical power to evaluate the association of PrEP exposure and uncommon pregnancy and infant outcomes. Additionally, most ongoing and planned studies are limited to women who initiate PrEP during pregnancy (Figure 2b), which will provide limited data on periconception or very early gestation PrEP exposure. Only in the MPYA and ZINK studies will women be on PrEP at the time of conception. The available data on women enrolled in PrEP efficacy trials who became pregnant provide some reassuring information on PrEP exposure during the early first trimester, when major organ development occurs 23. During the second trimester, drug exposures could affect foetal growth and brain development, yet no ongoing or planned studies will comprehensively evaluate neurocognitive outcomes in exposed infants. As PrEP becomes more available in HIV high‐burden settings and women receiving PrEP become pregnant or initiate PrEP at various times during pregnancy, it will be critical to monitor the timing of PrEP exposure and adverse pregnancy outcomes with sufficient sample sizes. Surveillance or contribution to birth registries such as the Antiretroviral Pregnancy Registry in settings with expanded PrEP use in young women may provide mechanisms to estimate risk of congenital abnormalities.

All ongoing or planned PrEP in pregnancy studies will evaluate pregnancy outcomes including gestational age at birth, birth length, birth weight, pregnancy loss and congenital abnormalities. None of study descriptions included power calculations for detecting differences in pregnancy outcomes. The field would benefit from well‐powered observational PrEP safety studies reporting perinatal outcomes that are based on calculations of the minimum detectable difference between exposure groups, including low, medium and high levels of TDF, to strengthen the interpretation of findings. IMPAACT 2009 is the only study that requires ultrasound prior to enrolment. Other studies rely on last menstrual period and clinician assessment of fundal height to determine gestational age at enrolment and subsequently gestational age at birth. Across studies, birth outcomes will be abstracted from clinical records, self‐reported by women at follow‐up visits, or recorded by clinical staff if births take place at study sites. When possible, gold standard measurements – such as first or early second trimester antenatal ultrasound – should be used for key outcomes to produce the highest quality safety data. This may be more difficult, however, when conducting studies in high HIV burden low‐resource settings with limited availability of technologies such as ultrasound and trained technicians.

#### 3.3.3 Outcomes beyond infancy among PrEP‐exposed children

Current ongoing studies will contribute perinatal outcome data from >6000 PrEP‐exposed pregnancies by 2022. However, we identified few studies evaluating prenatal PrEP exposure and longer‐term infant outcomes. In the SMARTT cohort, TDF use in pregnancy among HIV‐infected mothers was associated with reduced bone mineral content in neonates compared to exposure to other ARVs, though TFV concentrations in meconium were not associated with infant weight, length or bone mineral content 49, 50. A recent study using DXA suggests that prenatal TDF exposure in HIV‐exposed infants may lead to decrement in neonatal bone mineral density 51. One study of BMD among infants exposed to TDF‐containing ART found no association between duration of TDF exposure in utero and infant growth or BMD, though sample sizes in each exposure group were relatively small (<120 infants) and BMD was only measured very early in life (<21 days and 26 weeks). Evaluating BMD beyond infancy among larger samples of children prenatally exposed to TDF for PrEP could help bolster the safety evidence base given the BMD‐related concerns of TDF use 52. Further study is needed to confirm if these bone and growth differences persist beyond the neonatal period and if they are clinically relevant. Few studies identified in our review will evaluate infant outcomes beyond one year of life. Only two studies, CAP016 and IMPAACT 2009, will evaluate BMD among women who used PrEP while pregnant or breastfeeding. Other infant outcomes such as growth indicators will be measured in eight studies with follow‐up time ranging from six months to 1.4 years (Figure 2b). This highlights a need for studies on longer‐term outcomes beyond infancy and into early childhood following prenatal PrEP exposure.

#### 3.3.4 Outcomes among HIV‐uninfected women who use PrEP during pregnancy and/or lactation

The studies we identified included few maternal‐specific outcomes except for monitoring renal and liver function post‐PrEP initiation via routine laboratory tests. PrEP in pregnancy studies should include robust evaluation of mother‐focused safety outcomes beyond just pregnancy/infant outcomes. For example, three sub‐studies within PrEP efficacy trials found that unintended consequences of TDF‐based PrEP use include decline in maternal bone mineral density (BMD), which may be exacerbated during pregnancy or breastfeeding 53, 54, 55. Exposure to these agents during adolescence or early adulthood, critical periods for skeletal growth and when pregnancy frequently occurs in LMICs, could prevent the attainment of peak bone mass, a major determinant of increased fracture risk in later life 56. Additionally, bone metabolism alterations that occur during normal pregnancy and lactation lower BMD, which could result in greater decreased BMD when PrEP is used among pregnant and breastfeeding mothers. Only one study, IMPAACT 2009, will evaluate BMD among women who used PrEP while pregnant or breastfeeding. Future safety evaluations would be strengthened by including maternal bone health outcomes.

PrEP demonstration projects among young women from ongoing studies have found high rates of laboratory‐confirmed sexually transmitted infections (STIs) among young African women using PrEP 57. Complications of STIs in pregnancy include spontaneous abortion, stillbirth, preterm birth, and low birth weight 58. In PrIYA, researchers found that only 1% of pregnant women screened for PrEP recently had an STI diagnosed with syndromic algorithms, which have poor sensitivity and specificity for detection of STIs 59. This contrasts to the >30% prevalence of laboratory‐confirmed STIs (35% CT, 15% NG) detected among women screened for PrEP in PrEP demonstration projects among young women from the same region 57 These data suggest that an appreciable proportion of women who initiate PrEP in programmatic settings may have undiagnosed STIs. Prevalent STIs could confound the risk of adverse birth outcomes in PrEP studies, which could be more likely related to STIs and not PrEP. Evaluating STIs among maternal PrEP users is needed and only IMPAACT 2009 and the PrEP‐PP study (NCT#03902418) will include laboratory STI testing within the context of PrEP in pregnancy.

Our review did not include summarization of social science and qualitative studies related to PrEP use among pregnant and breastfeeding women. This is an important dimension that complements clinical trial evidence 60.

## 4 Conclusions

Expanding delivery of PrEP to young women of reproductive age and pregnant women at high risk of HIV acquisition is an essential strategy to reduce HIV incidence in pregnancy and breastfeeding women. Prior studies demonstrate that exposure to TDF and FTC as ART in pregnancy among women living with HIV is safe and well‐tolerated; limited studies of PrEP among pregnant women not living with HIV are also reassuring 18. Given the significant implications of acquiring HIV in pregnancy for both the woman and her child and the available safety data, the benefits of using PrEP for prevention of HIV acquisition in pregnancy and breastfeeding clearly outweigh potential risks and could substantially reduce maternal and infant HIV acquisition globally 7, 13, 15, 16, 61.

However, as PrEP in pregnancy research and programmes expand, there will also be an increase in the frequency of fetal PrEP exposure, and it is critical to augment the current data on maternal and infant safety. Future studies should better elucidate PrEP exposure within maternal and infant populations and evaluate implications of TDF drug levels for HIV protective efficacy in the context of pregnancy or the postpartum period. Pooling data across studies will enable more robust analyses of uncommon outcomes, though standardized assessment approaches are needed. Studies should also evaluate outcomes beyond infancy among children exposed to PrEP and woman‐focused safety outcomes following PrEP use during pregnancy and/or lactation. Planned studies will provide important new data on perinatal and infant outcomes following prenatal PrEP exposure, but research gaps remain that will be important to address as maternal PrEP programmes are implemented. Research and pharmacovigilance to provide more and longer‐term safety data and better quantification of amount and duration of PrEP exposure during pregnancy and breastfeeding are relevant to policymakers, programme implementers, providers, and pregnant and lactating women. As PrEP use in pregnancy burgeons, it is paramount to maximize opportunities to evaluate PrEP safety and develop a more complete body of evidence. In parallel, expanding PrEP delivery to pregnant and breastfeeding women at high risk of HIV is an essential prevention strategy.

## Competing interest

This paper represents the opinions of the authors and is not meant to represent the position or opinions of the organizations, including the World Health Organization, nor the official position of any staff members.

## Authors' contributions

DJD conceptualized the idea for the analysis, convened the initial meeting of the group, conducted the meta‐analysis, reviewed the data in the tables, wrote the first drafts, conducted the edits for the drafts, reviewed and approved final draft. JP conceptualized the idea for the analysis, reviewed articles for the meta‐analysis, reviewed the data in the tables, wrote components of the first draft, conducted the edits for the drafts, drafted the figures, reviewed and approved final draft. JB conceptualized the idea for the analysis, reviewed the data in the tables, revised first and second drafts, reviewed and approved final draft. RB, CC, BC, DM, LM, NM, RH, LTM, and LSC, reviewed data in tables/manuscript, reviewed drafts and provided substantial edits and contributed towards final draft, reviewed and approved final draft. LGB, JEH, JM, TC, LM, JK, GA, SS, and AM reviewed drafts and provided substantial edits and contributed towards final draft, reviewed and approved final draft. GJS conceptualized the idea for the analysis, reviewed the data in the tables, revised first and second drafts, reviewed and approved final draft.
